# Prospective Associations Between Intervention Components and Website Engagement in a Publicly Available Physical Activity Website: The Case of 10,000 Steps Australia

**DOI:** 10.2196/jmir.1792

**Published:** 2012-01-11

**Authors:** Cally Davies, Kelly Corry, Anetta Van Itallie, Corneel Vandelanotte, Cristina Caperchione, W Kerry Mummery

**Affiliations:** ^1^Centre for Physical Activity StudiesInstitute for Health and Social Science ResearchCQUniversity AustraliaRockhampton, QueenslandAustralia; ^2^Faculty of Health and Social DevelopmentUniversity of British ColumbiaKelowna, BCCanada; ^3^Faculty of Physical Education and RecreationUniversity of AlbertaEdmonton, ABCanada

**Keywords:** Physical activity, engagement

## Abstract

**Background:**

Effectiveness of and engagement with website-delivered physical activity interventions is moderate at best. Increased exposure to Internet interventions is reported to increase their effectiveness; however, there is a lack of knowledge about which specific intervention elements are able to maintain website engagement.

**Objective:**

To prospectively study the associations of website engagement and exposure to intervention components for a publicly available physical activity website (10,000 Steps Australia).

**Methods:**

Between June and July 2006 a total of 348 members of 10,000 Steps completed a Web-based survey to collect demographic characteristics. Website engagement was subsequently assessed over a 2-year period and included engagement data on website components; individual challenges, team challenges, and virtual walking buddies; and indicators of website engagement (average steps logged, days logging steps, and active users).

**Results:**

On average participants logged steps on 169 (SD 228.25) days. Over a 2-year period this equated to an average of 1.6 logons per week. Binary logistic regression showed that individuals who participated in individual challenges were more likely to achieve an average of 10,000 steps per day (odds ratio [OR] = 2.80, 95% confidence interval [CI] 1.45–5.40), log steps on a higher than average number of days (OR = 6.81, 95% CI 2.87–13.31), and remain an active user (OR = 4.36, 95% CI 2.17–8.71). Additionally, those using virtual walking buddies (OR = 5.83, 95% CI 1.27–26.80) and of older age logged steps on a higher than average number of days. No significant associations were found for team challenges.

**Conclusions:**

Overall engagement with the 10,000 Steps website was high, and the results demonstrate the relative effectiveness of interactive components to enhance website engagement. However, only exposure to the interactive individual challenge feature was positively associated with all website engagement indicators. More research is needed to examine the influence of intervention components on website engagement, as well as the relationship between website engagement and physical activity change.

## Introduction

Despite the well-documented health benefits associated with engaging in regular physical activity, over half of the people in Western countries are insufficiently active to receive health benefits [1]. Hence, there is a need for effective low-cost physical activity interventions that have a large reach. The Internet is a delivery medium that is able to incorporate interactive, specialized, and individualized health promotion tools to reach large populations at low cost, and is available anywhere and anytime [2,3]. Physical activity promotion programs delivered via the Internet have proven successful in producing short-term behavior change [4-12]. However, it is often reported that these types of interventions have high attrition and limited or declining website engagement [4,10,13-19]; and it is shown that low intervention exposure results in lower overall effectiveness of the intervention [2,16,17,20]. Hence, more research is needed to examine what components enhance website engagement and website use in order to increase their efficiency [14,16,21]. To date, efficacy studies have largely neglected to use objective engagement measures to examine specific intervention components that may increase website engagement [22]. Interactive website features (such as forums, regularly updated content, and online logbooks) have most often been suggested to enhance website engagement; however, there is little evidence to confirm this [22-24].

An existing, freely accessible physical activity promotion program that uses the advantages of Internet delivery to reach a large population is the Australian 10,000 Steps program. The 10,000 Steps program collects advanced website statistics, which allows the opportunity to prospectively examine and identify specific website components associated with website engagement. Studying a publicly available physical activity promotion website offers insight into how people use Internet-delivered programs in real life and can improve the ecological validity of these types of health behavior change programs. This is important, as data from real-life physical activity interventions are scarce, and findings from controlled clinical settings may not effectively translate to real-life settings. Therefore, the aim of the present study was to examine associations between exposure to intervention components and website engagement in a publicly accessible physical activity website (www.10000steps.org.au).

## Methods

### Participants

The participants were a sample of users of the Step Log feature on the existing 10,000 Steps website (www.10000steps.org.au). In June and July 2006, we randomly selected 663 registered participants from the 10,000 Steps program from a sample of over 24,000 Step Log users. Selected participants were current users, which was defined as having used the Step Log feature for at least 1 day during the month before the survey was conducted. In June and July 2006, we used email to approach potential participants and administer the Web-based demographic questionnaire.

### Procedures

The initial email contained an introductory letter outlining the purpose of the research, an invitation to participate, and the URL address, which led to a password protected Web-based questionnaire. By accessing the online questionnaire, participants gave informed consent to be part of the study. To enhance questionnaire completion, participants received three reminder emails to prompt survey completion. The first one was sent 4 days after the initial email, the second at 9 days, and the third 16 days after the original email [25].

Baseline measures of website engagement during May 2006 were extracted from the 10,000 Steps website for all participants who completed the Web-based questionnaire. Additionally, we monitored participants’ website engagement over a 2-year period following the completion of the baseline questionnaire. Engagement statistics were downloaded from the 10,000 Steps website 2 years after the baseline data collection (May 2008) for all participants who completed the Web-based questionnaire. A 2-year timeframe was chosen to assess long-term engagement with the website [4-6]. Unique website-user identification numbers allowed for the website statistics, over the 2-year period, to be matched for each individual participating in the survey. Prior to undertaking the study, we obtained ethical approval from the Human Research Ethics Committee at CQUniversity, Rockhampton, Australia.

### Intervention

The 10,000 Steps program was initially developed as a multilevel, multistrategy program targeted to the adult population to increase physical activity levels. The program was first delivered in Rockhampton, Australia as a whole-community program based on the socioecological framework [26]. A key aspect of the program is the use of the pedometer to record and monitor physical activity. The use of a pedometer is closely aligned with the prescriptive nature of the program’s name, which encourages the accumulation of physical activity in terms of steps per day [26]. Further background on the conceptualization and development of the program has been reported elsewhere [27]. Based on the success of the program [28,29] the funding body, Queensland Health, provided ongoing funding to continue the development, dissemination, and assessment of the program.

**Figure 1:**
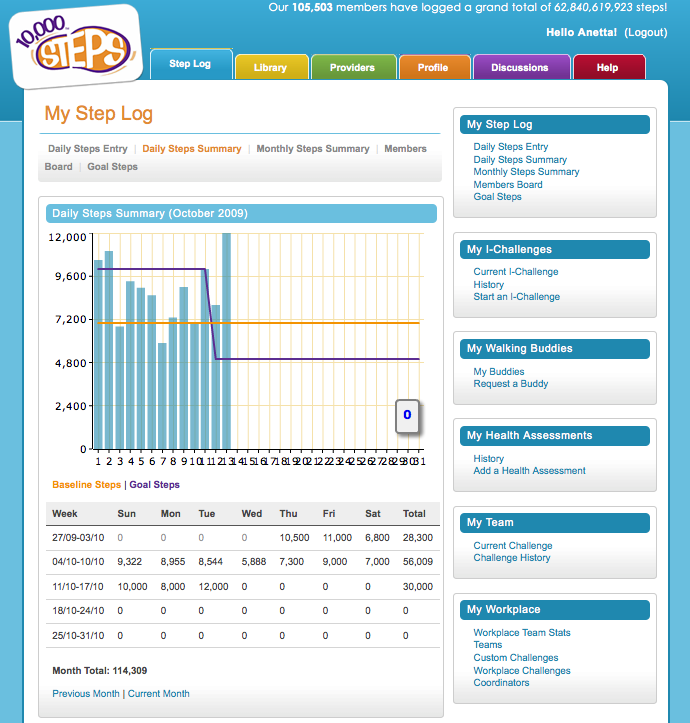
Screenshot of the online Step Log overview

**Figure 2:**
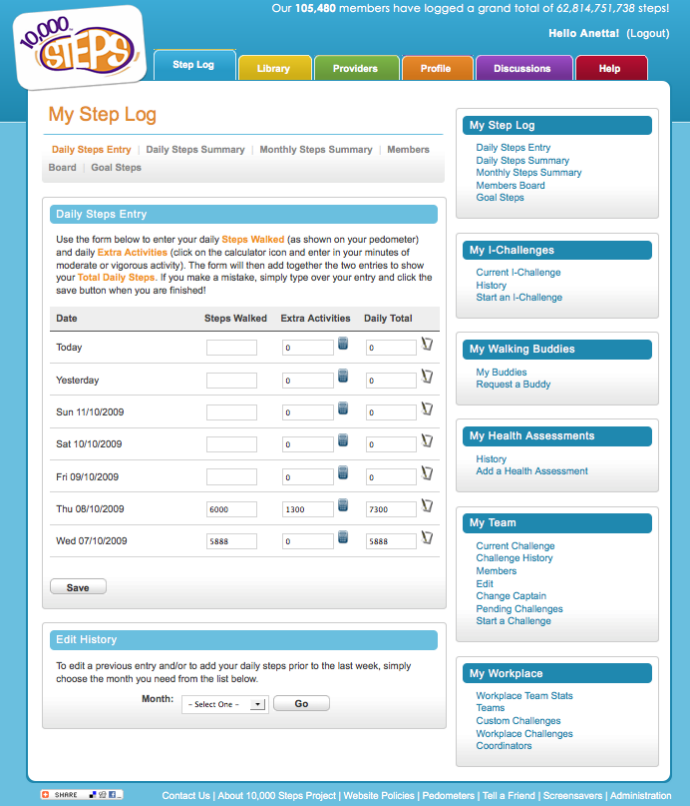
Screenshot of the online Step Log daily steps entry

**Figure 3:**
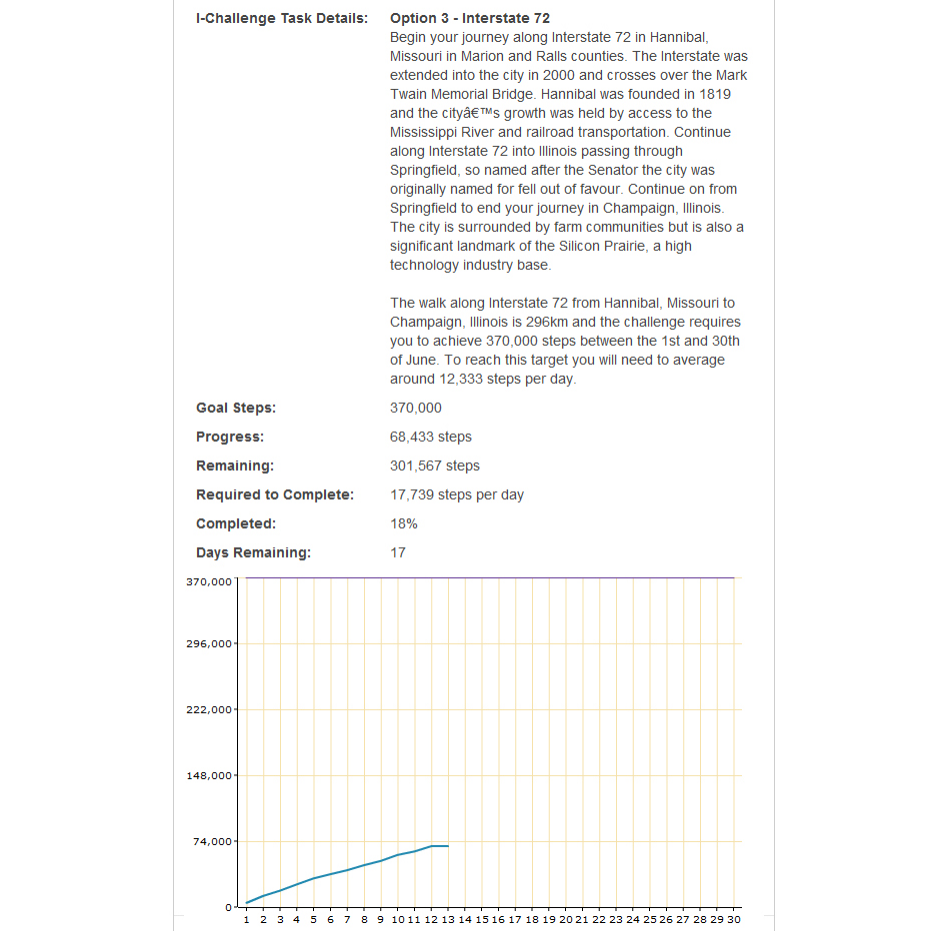
Screenshot of the I-Challenge feature.

 This funding supported the development of the 10,000 Steps website, which houses all of the resources and materials for different user groups to implement the 10,000 Steps program in their chosen setting (eg, workplaces). In addition to these resources the website includes an online Step Log (Figure 1), which allows and encourages participants to record and monitor daily physical activity levels in the form of steps (recorded from a pedometer) or time spent in moderate and vigorous physical activity (Figure 2), or both. The 10,000 Steps online Step Log also contains additional interactive components. For example, the individual challenges (I-challenges*)* allow individuals to choose from a selection of predefined monthly goals (actual steps walked) that correspond to a virtual walking challenge; a new challenge is available each month (Figure 3). Team challenges allow workplaces to offer staff members the opportunity to participate in a virtual walking challenge as part of a team. Additionally, any registered Step Log member also has the option to add virtual walking buddies, with whom they can share progress through the use of the Step Log. As of July 2011 the 10,000 Steps program had over 160,991 registered participants, who have logged a total of 86,528,244,202 steps.

### Measures

#### Demographic

The following demographic information was collected from the baseline survey: gender, age category, education level, household income (Australian dollars), body mass index, and presence of a chronic disease. These questions were based on surveys used by the Australian Bureau of Statistics [30].

#### Website Statistics

##### Baseline Engagement

As participants in the current study were already using the 10,000 Steps website before the demographic questionnaire was distributed, we extracted 3 baseline engagement measures from the website for May 2006. These measures included the number of months participants had been members of the 10,000 Steps website prior to May 2006; the average number of steps logged on the 10,000 Steps website per Step Log day in May 2006; and the number of days that steps were recorded on the Step Log during May 2006.

##### Exposure

Exposure to specific website components included use of 3 major interactive features within the 10,000 Steps website (team challenges, I-challenges, and virtual walking buddies). These statistics were used as independent measures to prospectively assess website engagement. Outcomes were dichotomized into a yes-or-no format, indicating the use of the feature on one or more occasion within the 2-year period.

##### Engagement

Website engagement was expressed through three website engagement variables: average steps, Step Log days, and last Step Log date. These outcome measures were dichotomized to allow for binary logistic regression as follows: average steps per logged day over the 2-year period was dichotomized into (1) participants who on average recorded 10,000 steps per day or more, and (2) participants who on average recorded less than 10,000 steps per day. The average Step Log days over the 2-year period was dichotomized based on the mean split into (1) participants who equaled or exceeded the mean number of days that steps were logged during the 2-year period across participants, and (2) participants who were below this mean. Finally, the last Step Log date for each participant was used to determine whether participants were still using the website. Participants were dichotomized into (1) users who logged steps on or after January 1, 2008 (active users), and (2) nonusers whose last Step Log date was prior to January 1, 2008.

##### Website Statistics Rationale

Objective website engagement statistics were measured in 2 categories: exposure to website components and website engagement. Use of the 3 interactive features mentioned above were conceptualized to provide measures for exposure to intervention components. In addition to being 3 of the main components in the 10,000 Steps website, they also facilitate behavior change through theoretical elements such as self-monitoring, goal-setting, feedback, and social support. Therefore, exposure to intervention components was conceptualized as use of the I-challenges, team challenges, or virtual walking buddies over a 2-year period as described below. We hypothesized that exposure to these intervention components would increase website engagement.

We chose 3 separate measures to provide a comprehensive overview of website engagement: average steps per logged day, Step Log days, and the last Step Log date. Each of these measures provides slightly different information about website engagement. First, we conceptualized average steps per logged day as a measure of website engagement; it should not be considered as an outcome measure, as the steps logged on the website did not encompass overall physical activity levels, and we did not measure any other form of physical activity. The Step Log is an intervention feature to benefit participants; it is not a measurement tool. The second measure, Step Log days, is most in line with the norm for measuring website engagement (being number of website logins) but could be considered to be a more in-depth measure, as participants not only log in to the website but also record steps taken. The final measure is last Step Log date. A cut-off date close to the end of the 2-year observational period was chosen (January 2008) to be able to classify participants as current users and therefore currently engaged with the 10,000 Steps program. These 3 forms of website engagement measurements combine to provide an overall view of website engagement and allow for the thorough investigation of associations between exposure to intervention components and website engagement.

### Statistical Analyses

We analyzed descriptive statistics of participant characteristics and website engagement. Binary logistic regression analyses were undertaken to prospectively examine the crude and adjusted odds ratios (ORs) for average steps, Step Log days, and last Step Log date. Independent variables assessed included participant demographics and exposure to the specific website components (team challenges, I-challenges, and virtual walking buddies). First, we determined crude ORs for all three dependent variables (average steps logged, Step Log days, and last Step Log date). Next we calculated adjusted ORs for significant crude ORs with the addition of gender, age group, education, income, chronic disease status, number of months of membership prior to May 2006, average steps recorded in May 2006, and number of days steps were logged during May 2006. With the exception of the variables listed, only variables that displayed significant crude ORs were included in the adjusted model; all other variables were excluded, as they did not have a statistically significant effect in the initial model [31]. Statistical analysis was undertaken using SPSS version 17.0 (IBM Corporation, Somers, NY, USA), with the significance level being set to *P* < .05.

## Results

### Demographics

Of the 663 emails distributed, 15 were undeliverable to addresses used and 300 were not responded to after three prompts, resulting in 348 participants completing the Web-based questionnaires (52.5%). The majority of participants were female (196/306, 64.1%); between 45 and 54 years old (121/306, 39.5%); held a university degree or higher (157/310, 50.7%); and earned under A$75,001per year (157/303, 51.8%). Table 1 presents a descriptive summary of participant characteristics, also broken down by website engagement statistics.

**Table 1:** Descriptive summary of participant characteristics by website engagement statisticsa

Characteristic				% of average steps >10,000/day		% of Step Log days above average		% of Step Log users in January 2008	
		n	%	n	%	n	%	n	%
Total		348		161	46.3	97	28	66	19
**Gender (n = 306)**									
	Male	110	35.9	63	57	38	35	23	21
	Female	196	64.1	83	42	52	27	36	18
**Age group (years) (n = 306)**									
	18–34	62	20	27	44	8	13	10	16
	35–44	77	25	33	43	19	25	12	16
	45–54	121	39.5	58	48	39	32	23	19
	≥55	46	15	29	63	25	54	15	33
**Education (n = 310)**									
	≤ Year 10	29	9	12	41	6	21	4	14
	Year 12	37	12	21	57	10	27	7	19
	TAFE^b^ certificate/diploma or equivalent	87	28	46	53	32	37	20	23
	University degree or higher degree	157	50.6	69	44	44	28	30	19
**Household income (A$) (n = 303)**									
	Nil–52,000	93	31	48	52	26	28	16	17
	52,001–75,000	64	21	31	48	17	27	15	23
	75,001–100,000	42	14	23	55	15	36	7	17
	>100,000	62	21	30	48	21	34	13	21
	No response	42	14	12	29	11	26	9	21
**Body mass index category (kg/m^2^) (n = 280)**									
	<25	110	39.2	55	50	33	30	25	23
	25–29.9	106	37.9	55	52	37	35	23	22
	≥30	64	23	29	45	16	25	11	17
**Chronic condition (n = 310)**									
	Yes	60	19	27	45	28	47	19	32
	No	250	80.6	121	48.4	63	25	42	17

^a^ Data collected at baseline (June and July 2006).

^b^ Technical and Further Education.

### Objective Website Statistics

#### Baseline Engagement

The mean length of membership before the demographic questionnaire was distributed for the sample was 4.88 (SD 5.02) months. The average number of steps logged per Step Log day during May 2006 was 11,295 (SD 4391.47) and the average days that steps were logged in May 2006 was 22 (SD 11.22).

#### Exposure

Website engagement data show that the team challenges demonstrated the highest use (137/348, 60.6%), whereas only a minority of participants used the I-challenge or had virtual walking buddies (29.3% and 4.3% respectively; Table 2).

**Table 2:** Descriptive summary of objective website statisticsa

Characteristic		n	%	% of Average Steps >10,000/day	% of Step Log days above average	% of current users in January 2008
**Team challenge (n = 348)**						
	Yes	211	60.6	45.5	23.2	17.5
	No	137	39.4	47.4	35	21.5
**Individual challenge (n = 348)**						
	Yes	102	29.3	60.8	53.9	35.3
	No	246	70.7	40.2	17.1	12.2
**Virtual walking buddy (n = 348)**						
	Yes	15	4	60	66.7	33.3
	No	333	95.7	45.6	26.1	18.3

^a^ Data show website engagement from June 2006 to May 2008.

#### Engagement

For the indicators of website engagement, average steps per logged day during the 2-year period was 9507 (SD 6665), with 46.3% (161/348) of participants achieving an average of more than 10,000 steps per day for the entire period between 2006 and 2008 (Table 1). Participants reported an average of 169 (SD 228.25) Step Log days, with 28% (97/348) of participants classified as being above the average split. Finally, 19% (66/348) of the participants were still using the 10,000 Steps website in 2008, 20 months after baseline.

### Determinants of Website Engagement

The adjusted logistic regression model (Table 3) showed that participants who were male (OR = 1.12, 95% CI 1.13–3.97) or who participated in I-challenges (OR = 2.80, 95% CI 1.45–5.40) were significantly more likely to report average steps above 10,000 per logged day. Participants who were older, participated in I-challenges (OR = 6.18, 95% CI 2.87–13.31), or had virtual walking buddies (OR = 5.83, 95% CI 1.27–26.80) were significantly more likely to log above the average Step Log days. Finally, individuals who participated in an I-challenge (OR = 4.36, 95% CI 2.17–8.71) were significantly more likely to remain active users of the 10,000 Steps website than those who did not.

**Table 3:** Adjusted odds ratios for website engagement

Characteristic		Adjusted OR^a^ (95% CI^b^) average steps >10,000/day	Adjusted OR (95% CI) Step Log days above average (169)	Adjusted OR (95% CI) current users in January 2008
**Gender (n = 306)**				
	Female	1^c^	1	1
	Male	1.12 (1.13–3.97)^d^	1.76 (0.84–3.70)	1.23 (0.61–2.45)
**Age group (years) (n = 306)**				
	18–34	1	1	1
	35–44	0.96 (0.41–2.25)	2.70 (0.88–8.33)	0.99 (0.37–2.68)
	45–54	1.20 (0.54–2.65)	3.02 (1.01–9.04)^d^	1.01 (0.40–2.57)
	≥55	2.25 (0.79–6.36)	9.74 (2.75–34.59)^d^	1.88 (0.62–5.71)
**Education (n = 310)**				
	≤ Year 10	1	1	1
	Year 12	0.69 (0.24–1.99)	0.50 (0.14–1.85)	0.81 (0.23–2.90)
	TAFE^e^ certificate/diploma or equivalent	1.59 (0.60–4.18)	0.72 (0.22–2.34)	0.89 (0.30–2.60)
	University degree or higher degree	0.85 (0.42–1.70)	1.20 (0.52–2.80)	0.97 (0.45–2.07)
**Household Income (A$) (n = 303)**				
	Nil–52,000	1	1	1
	52,001–75,000	0.72 (0.31–1.69)	0.55 (0.19–1.57)	1.61 (0.64–4.08)
	75,001–100,000	0.92 (0.36–2.37)	0.95 (0.32–2.88)	0.85 (0.27–2.66)
	>100,000	0.66 (0.29–1.54)	0.95 (0.34–2.68)	1.23 (0.47–3.23)
	No response	0.42 (0.15–1.14)	0.73 (0.23–2.31)	1.61 (0.56–4.64)
**Chronic condition (n = 310)**				
	No	1	1	1
	Yes	0.55 (0.25–1.19)	1.94 (0.80–4.78)	1.44 (0.64–3.24)
**Team challenge (n = 348)**				
	Yes		1	
	No		1.07 (0.52–2.19)	
**Individual challenge (n = 348)**				
	No	1	1	1
	Yes	2.80 (1.45–5.40)^d^	6.18 (2.87–13.31)^d^	4.36 (2.17–8.71)^d^
**Virtual walking buddy (n = 348)**				
	No		1	
	Yes		5.83 (1.27–26.80)^d^	

**Baseline engagement**				
	Length of membership^f^	1.07 (1.01–1.14)^d^	1.21 (1.13–1.29)^d^	1.08 (1.02–1.15)^d^
	Average steps^g^	1.00 (1.00–1.00)^d^	1.02 (0.98–1.01)	0.98 (0.99–1.00)
	Average Step Log days^g^	0.98 (0.95–1.01)	1.05 (1.01–1.09)^d^	0.99 (0.96–1.02)

^a^ Odds ratios (OR) mutually adjusted for all other significant crude OR variables and chronic disease status, gender, age, education, household income, length of membership, average steps in May 2006, and Step Log days in May 2006.

^b^ Confidence interval.

^c^ Reference category.

^d^ Significant OR values; only significant crude OR included and reported in adjusted OR model.

^e^ Technical and Further Education.

^f^ Length of membership in months prior to May 2006.

^g^ Averages observed during May 2006.

## Discussion

The primary aim of the present study was to examine associations between exposure to website components (such as interactive features) and website engagement in the Australian 10,000 Steps website (www.10000steps.org.au). This study is unique in that it prospectively examined engagement with a physical activity promotion website over a 2-year period. To our knowledge such an examination has not been published before, despite the demonstrated need to make physical activity promotion websites more effective in terms of behavior change and maintenance [2]. The results show that exposure to interactive intervention components can enhance website engagement in a publically accessible physical activity website in a sample of users who were already actively using the website at baseline. Specifically, exposure to the interactive I-challenge feature was associated with all indicators of website engagement during the 2-year observation period. Additionally, exposure to the virtual walking buddies feature was positively associated with recording above-average Step Log days.

I-challenges are one of the major interactive features incorporated in the 10,000 Steps website; participants choose their own physical activity goal for the month from a set of predefined virtual walking journeys and are able to view their progress through graphs and feedback text. The I-challenge feature was used by only 29.3% of participants, whereas 60.6% used the team challenges feature. Although I-challenges are similar to the team challenges, only exposure to the I-challenge was significantly associated with website engagement. This might be explained by the two prominent differences between these features. Individuals participating in the I-challenges choose to participate entirely of their own accord, whereas individuals participating in a team challenge are recruited by their workplace. This reflects a different motivation to become a 10,000 Steps member and may reflect why participants using the I-challenge feature are more likely to stay engaged with the website [32]. Additionally, new short-term I-challenges are created every month, which allow participants to progressively increase or adjust their goal steps each month. The I-challenge feature also allows individuals to choose from a selection of predefined monthly step goals that cater to different levels of physical activity, whereas the workplace is responsible for setting the overall goal for the team challenges (which in most cases are longer than 1 month). Research highlights the importance of setting personalized goals for ongoing self-management of behavior and to help facilitate behavioral change [33].

The present study reported high levels of program engagement in comparison with previous studies [4,14,21,34], as participants logged an average of 169 (SD 228.25) Step Log days over the 2-year period. This equates to 1.6 logons per week over the 2-year period, which is especially notable, as most previous research has demonstrated lower levels of program engagement over shorter study durations [4,14,21,35]. Marshall et al [35] found that only 26% of participants logged on to their study’s website more than once. McKay et al [4] reported an average of 1.1 logins per week throughout their 8 week Internet-delivered physical activity intervention within a sample of individuals with a diagnosis of type 2 diabetes. Steele et al [21] reported an average of 11.8 logins (0.98 logons per week) over the 12-week duration of their physical activity intervention in the general population. Using a longer study design, Lewis et al [36] reported a median of 44 logins (0.86 logons per week) over 12 months for their physical activity intervention. Consistent with the present study, the three studies reporting greater website engagement also incorporated the use of interactive features, which has been suggested to enhance website engagement [5]. This is important, as it has been shown that higher exposure to website content is related to greater behavior change levels [13,37].

Two additional reasons could be associated with the high level of website engagement we observed. First, during the initial 10,000 Steps program (2001–2003), emphasis was placed on marketing 10,000 Steps as a brand [26]. The initial program, a multistrategy community-based intervention, which has been described elsewhere [27], included an overarching marketing campaign. The marketing campaign established high brand recognition and awareness of the 10,000 Steps program that is still being seen today [38]. Since 2004, the 10,000 Steps resources developed in the initial program have been disseminated via the website [26]. By recognizing 10,000 Steps as a brand, participants might be more likely to perceive the website as credible. Website credibility is important for participant engagement, as previous research has shown that users prefer websites that contain credible content [39]. Second, through evaluations of the program, the 10,000 Steps website has been shown to have high levels of usefulness and usability [40], which have previously been shown to be an important factor for increased website engagement [36].

We found that both older age and the presence of virtual walking buddies were related to recording above the average Step Log days. Participants aged 45 years and older were significantly more likely than those aged between 18 and 34 years to engage with the 10,000 Steps program for a longer period of time. The same has been observed in other research [16,18]. Older people may place more importance on their health than younger people do, have more to gain, and are more at risk of developing or already have chronic disease. Additionally, as suggested in previous research, older adults may have more free time at their disposal in contrast to younger adults and as such may be more willing to allocate time to participating in physical activity programs, especially Internet-based programs [41].

The virtual walking buddies feature allows 10,000 Steps members to invite known members to be virtual walking buddies, and they can then view and compare each other’s physical activity progress. The positive impact of the virtual walking buddies on website engagement is not surprising, as previous studies have highlighted the importance of social support for behavior change [22,42]. Unfortunately, only 4% of participants made use of the virtual walking buddies in this study. This low number may be a limitation of the 10,000 Steps Step Log, which requires members to know the email address of the person they wish to add as a buddy. More research is needed to explore the effectiveness of virtual walking buddies, as well as how more participants could be encouraged to use it. Virtual social support platforms are becoming increasingly prevalent on the Internet, and exploring the application of virtual social support in relation to health promotion programs is an area of potential future research.

### Study Strengths and Limitations

It is important to recognize that there are both limitations and strengths associated with the present study. First, the study incorporates a selective and motivated sample. Included participants are from a sample who self-selected to register for the 10,000 Steps program and who voluntarily elected to respond to the questionnaire. The outcomes of this study might therefore not be entirely comparable with studies that report outcomes under controlled circumstances. Additionally, participants were already engaged with the website prior to the observation period. However, to account for this limitation, length of membership and baseline website engagement were adjusted for in the final model. Furthermore, due to our recruitment method, our study sample might not accurately reflect the demographic characteristics of all users of the 10,000 Steps website. In comparison with previously published statistics for age and gender for all registered members, as of March 2006 the average age was 40.46 (SD 11.74) and 66% were female, compared with an average age of 44.6 (SD 11.74) and 64% female in the current sample [26]. To compare website engagement for the current sample with all users of the 10,000 Steps program, we extracted website engagement statistics for all 10,000 Steps members. Website engagement data for all 10,000 Steps members from the commencement of the website in 2004 to July 2011 demonstrated an average of 12,237 steps per Step Log day and 59.7 Step Log days. Second, the present study did not measure physical activity behavior but instead focused on website engagement. It was not the purpose of the current study to investigate website engagement in relation to physical activity behavior, but rather to examine associations of exposure to interactive website components and website engagement in a physical activity website. Finally, only 19% of participants remained active users of the Step Log in January 2008. This places limitations on the generalizability of the study results to the general population. However, a significant strength of this unique study is the ability to record objective engagement statistics in a publicly accessible (real-life) health promotion website in order to gain a greater understanding of demographic and interactive features that influence website engagement.

### Conclusions

This study provides support for the use of a freely available health behavior change program and highlights the importance of including interactive features to enhance program engagement in a sample of individuals already engaged in a Web-based physical activity program. As of July 2011 the 10,000 Steps website had 160,991 registered users, who had logged a total of 86,528,244,202 steps; hence, the potential public health impact of Internet-delivered health promotion programs should not be underestimated. The findings suggest that exposure to interactive intervention components can enhance website engagement. In this particular physical activity promotion program, exposure to the I-challenges and to a lesser extent the virtual walking buddies feature were associated with higher engagement. Engagement in health promotion programs has previously been associated with increased behavior change. In this context higher engagement may lead to increased steps, which are linked to improved health outcomes. Future research should aim to examine the influence of specific intervention components on website engagement, as well as the relationship between website engagement and physical activity behavior change.

## Abbreviations

CI: confidence interval
I-challenge: individual challenge
OR: odds ratio
